# Supplementary material for: Pressure-driven dome-shaped superconductivity and electronic structural evolution in tungsten ditelluride
Source: Nat Commun. 2015 Jul 23;6:7805. doi: 10.1038/ncomms8805 (PMC4525151; doi:10.1038/ncomms8805)
Supplement: Supplementary Information — Supplementary Figures 1-5, Supplementary Tables 1-4, Supplementary Note 1 and Supplementary References [file ncomms8805-s1.pdf]

Supplementary Figures

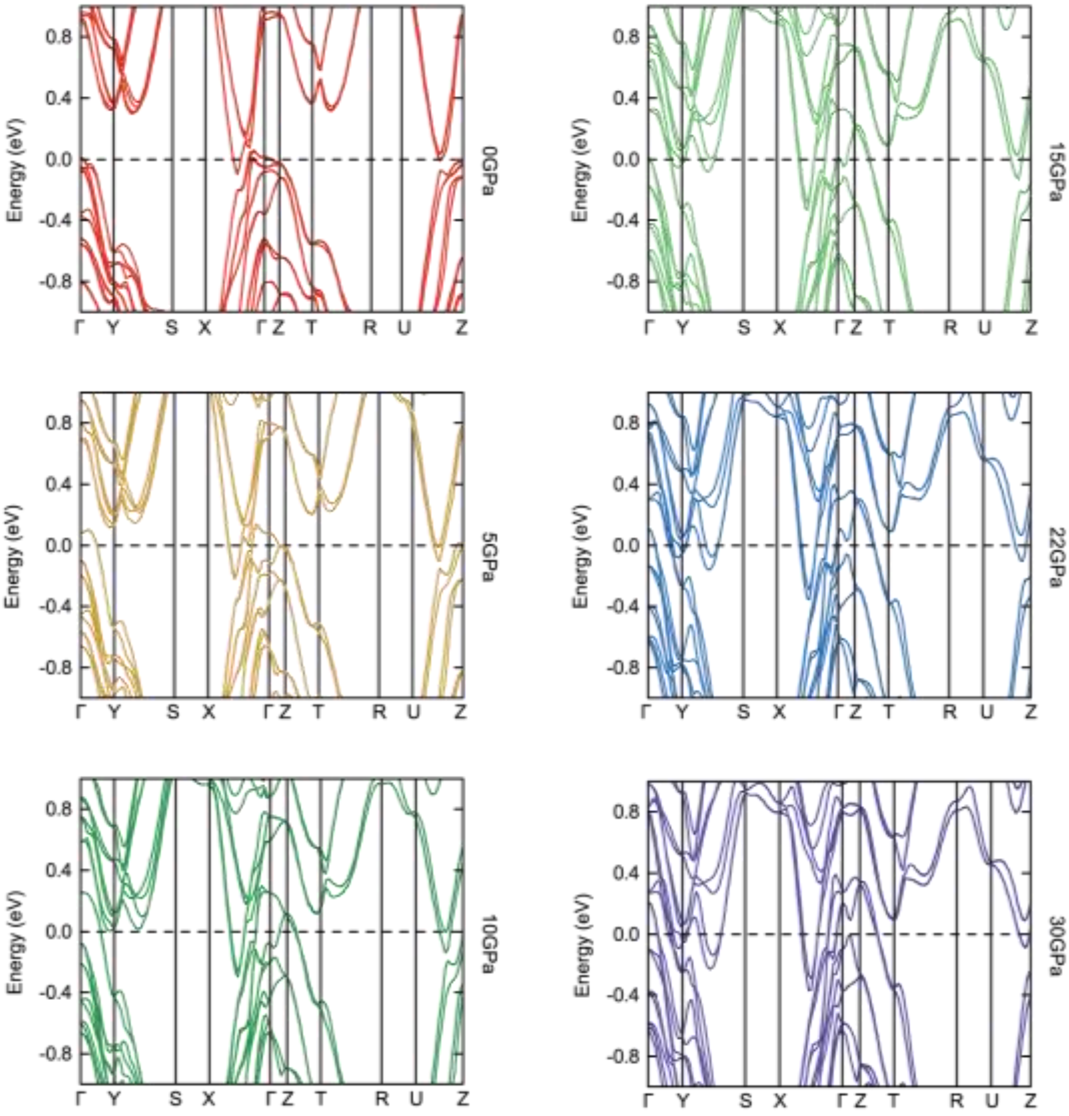

Supplementary Figure 1. Band structure as a function of pressure

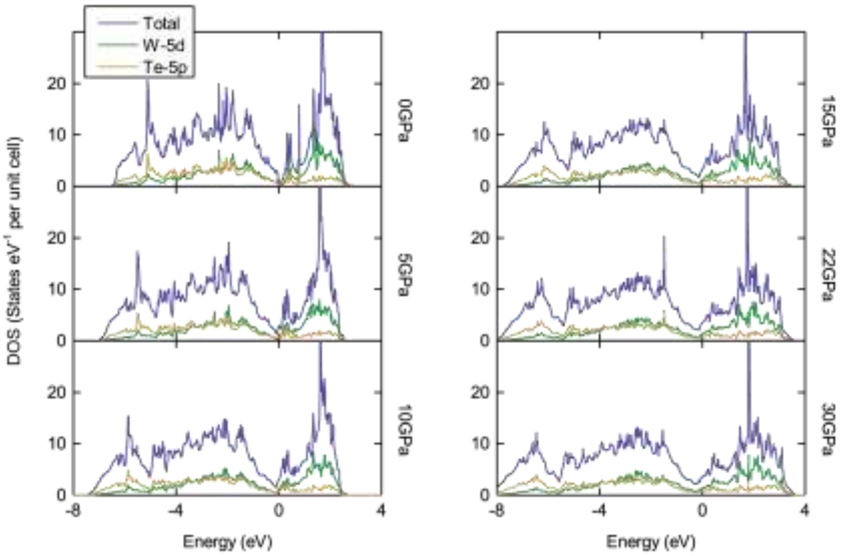

Supplementary Figure 2. Numerical density of states for different pressures

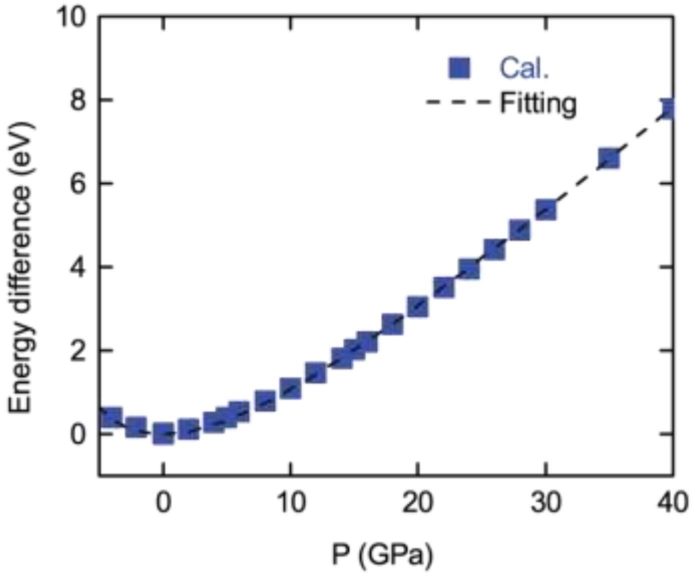

Supplementary Figure 3. Total energy as a function of pressure

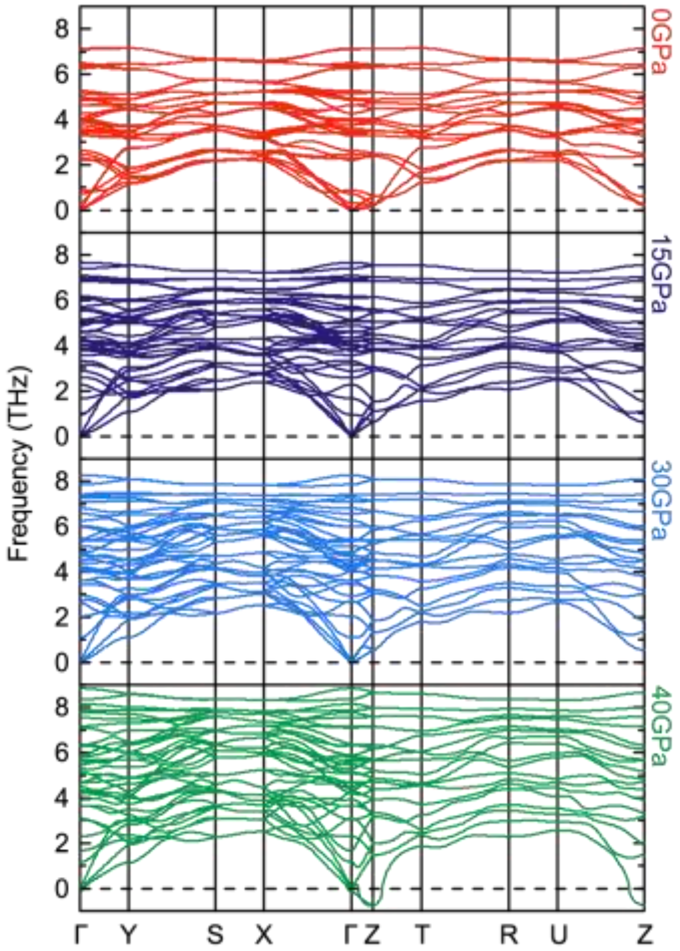

Supplementary Figure 4. calculated phonon spectra at various pressures

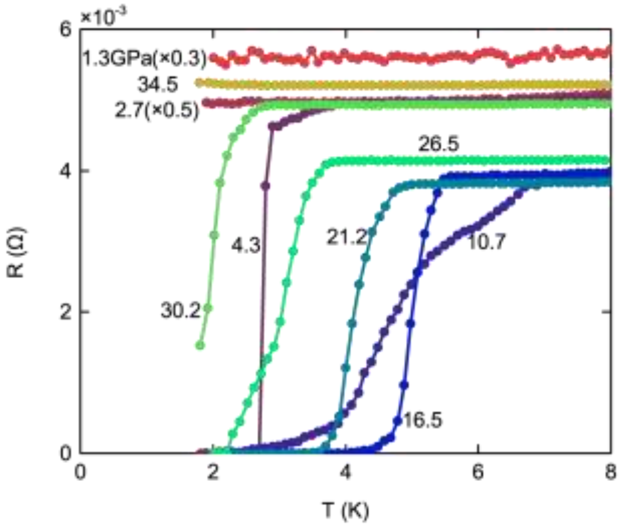

Supplementary Figure 5. The R-T measurements in run No. 3. The temperature-dependent resistance under different pressures up to 34.5 GPa in run No.3 are shown, where the superconductivity is induced at 4.3 GPa.

Supplementary Tables

**Formula**	**WTe_2**
**formula mass, amu**	439.05
**space group**	C_{2v}^7-$Pmn2_1$
**a, Å**	3.4859(19)
**b, Å**	6.265(3)
**c, Å**	14.038(8)
**α, °**	90.000
**β, °**	90.000
**γ, °**	90.000
**V, $Å^3$**	306.6(3)
**Z**	4
**$T_{Measurement}$, K**	296(2)
**$R(F^2)$**	0.0887
**$R_w(F^2)$**	0.2210

Supplementary Table 1. Crystal Data for WTe_2

**Atom**	**X**	**Y**	**Z**
**W(1)**	1.0000	0.4007(4)	0.61651(16)
**W(2)**	0.5000	0.0421(4)	0.60189(17)
**Te(1)**	1.0000	0.1446(6)	0.4630(3)
**Te(2)**	0.5000	0.6495(6)	0.5067(3)
**Te(3)**	1.0000	0.7950(7)	0.7148(3)
**Te(4)**	0.5000	0.2988(7)	0.7583(3)

Supplementary Table 2. Positional Parameters for WTe_2

Raman mode	Our(Cal.) (cm^{-1})	Ref.1 (Exp.) (cm^{-1})	Ref.2 (Exp.) (cm^{-1})
A_1^{10}	73.7		78.9
A_2^{5}	87.4		88.4
A_2^{4}	110.0	109.8	109.9
A_1^{9}	116.3	119.6	114.6
A_1^{8}	131.1	130.4	129.9
A_1^{6}	134.4	139.1	
A_1^{5}	161.8	160.5	160.6
A_1^{3}	208.8		207.7
A_1^{2}	218.2	208.4	

Supplementary Table 3. Comparison of our numerical phonon frequencies and experimental Raman results.

**Pressure (GPa)**	0	5	10	15	20	26	30
**V(10^6m/s)**	0.59	1.37	1.72	1.96	2.13	2.27	2.34

Supplementary Table 4. Numerical Fermi velocity as a function of pressure

Supplementary Note 1

For the optimized crystal structure, we performed a phonon calculation. A unit cell contained twelve atoms, resulting in 36 phonon branches in a phonon dispersion curve. As shown in the supplementary Figure 4, the overall shape of our numerical phonon dispersion at zero pressure is consistent with that obtained from density perturbation functional calculations [1]. As shown in the supplementary Table 4, our numerical phonon frequencies agree with the experimental results [1, 2]. As shown in the supplementary Figure 4, at zero pressure, the phonon branches have only slight dispersion along the Γ-Z direction, which again indicates the layered nature of this compound. Analysis of the evolution of the phonon eigenvectors in the Brillouin zone shows that Te-W vibrations are strongly coupled and extend over the whole frequency region. Because the space group possesses non-primitive translation properties of ($\vec{\tau} = \frac{\mathrm{a}}{2}\vec{i} + \frac{\mathrm{c}}{2}\vec{k}$), the modes along the Brilliant Zone broader lines (S-X, Z-T-R-U-Z) are twofold degenerate, while due to the lattice distortion and low crystal symmetry, most of the phonon modes are non-degenerate, and there are no pure in-plane or out-of-plane vibration modes. This is in sharp contrast to more common TMDs such as 2H-MoS_2, for which there exist two-degenerate vibration modes (E^1_{2g} and E^2_{2g}) with definite in-plane characteristics [3].

We also investigated the pressure-induced phonon evolution, and show several representative phonon dispersions in the supplementary Figure 4. Phonon dispersions under different pressures share a resemblance to that at zero pressure, and consistent with the variation in the *c*/*a* ratio, the dispersion along Γ-Z direction increases with

increasing pressure as shown in the supplementary Figure 4. The shrinking of the lattice parameter with increasing pressure also results in higher phonon frequencies. A striking feature of this phonon spectrum as shown in the supplementary Figure 4 is a possible structural instability under compression as indicated in the presence of soft phonon modes at 40 GPa.

Supplementary References

[1] Jiang, Y. C. & Gao, J. Raman fingerprint of semi-metal WTe_2 from bulk to monolayer. Preprint at http://arxiv.org/abs/1501.04898 (2015).

[2] Kong W-D, et al. Raman scattering investigation of large positive magnetoresistance material WTe_2. Applied Physics Letters **106**, 081906 (2015).

[3] C. Lee, H. Yan, L.E. Brus, T.F. Heinz, J. Hone and S. Ryu, Anomalous Lattice Vibrations of Single- and Few-Layer MoS_2. ACS Nano **4**, 2695-2700 (2010).
